# Regional metabolomic profiling reveals lipid metabolic signatures associated with oil content in flue-cured tobacco

**DOI:** 10.3389/fpls.2026.1734410

**Published:** 2026-03-16

**Authors:** Guanhui Li, Qi Xu, Zeyu Zhao, Kesu Wei, Jiati Tang, Xueyan Jing, Cheng Zhang, Chenghong Shen, Yingchao Lin, Xuekun Zhang, Benbo Xu, Shengjiang Wu

**Affiliations:** 1 College of Agriculture, Yangtze University, Jingzhou, China; 2 Guizhou Academy of Tobacco Science, Upland Flue-Cured Tobacco Quality & Ecology Key Laboratory of China Tobacco, Guiyang, China; 3 Zunyi Tobacco Company of Guizhou Province, Zunyi, China

**Keywords:** flue-cured tobacco, oil content, regional metabolism, ultrastructure, widely targeted metabolomics

## Abstract

**Introduction:**

Flue-cured tobacco (*Nicotiana tabacum*) leaves exhibit substantial variation in oil content, yet the metabolic basis underlying these differences remains unclear.

**Methods:**

In this study, we integrated physicochemical measurements, ultrastructural observation, and metabolomic profiling to investigate oil-associated metabolic characteristics. High-oil and low-oil leaves from Tongren (TR; TRH, high-oil; TRL, low-oil) and Weining (WN; WNH, high-oil; WNL, low-oil) were analyzed.

**Results:**

High-oil leaves exhibited higher petroleum ether extract content (TRH 5.56%; TRL 4.63%; WNH 4.82%; WNL 4.15%) and lower softness value (TRH 34.7 mN; TRL 47.3 mN; WNH 21.7 mN; WNL 36.3 mN), with reduced luminosity (L*) and increased red-green axis (a*). Ultrastructural analysis revealed a greater abundance of lipid droplets in high-oil leaves, while starch granules persisted in low-oil leaves from TR. Metabolomic profiling identified 38 differential metabolites in TR (upregulated: triacylglycerols, phospholipids, fatty acid derivatives; downregulated: polyphenols, phenylpropanoids) and 47 in WN (upregulated: flavonoids, coumarins, sesquiterpene lactones; downregulated: organic acids, sugar alcohols, some fatty acid derivatives). Four metabolites—namely, marmesin, an arteannuin-like compound, isoferulic acid, and p-hydroxycinnamic acid—were found to be prevalent in both regions and exhibited a significant correlation with oil-related traits.

**Discussion:**

Pathway enrichment analysis further indicated that high-oil leaves from TR and WN displayed distinct metabolic patterns despite similar oil phenotypes. These findings suggest that oil accumulation is associated with region-distinct metabolic profiles and provide potential metabolic markers for oil-related quality improvement.

## 1 Introduction

Tobacco (*Nicotiana tabacum*), a key member of the Solanaceae family, holds significant economic value and has long served as an important model for studying plant physiology, biochemistry, and metabolic regulation. The insights derived from tobacco, particularly with regard to lipid metabolism, can prove to be highly informative for other Solanaceous crops. Flue-curing of tobacco leaves is essentially a dehydration–thermal processing procedure conducted under precisely controlled artificial conditions. Its primary objective is not merely water removal, but the application of a controlled and directed stress to living plant tissues through the deliberate design of temperature and moisture trajectories. This process induces a series of physiological events, including programmed cell death, macromolecular degradation, and complex interactive chemical reactions, ultimately converting fresh leaves into an industrial raw material with distinctive sensory attributes (Wu et al., 2020a). Studies on the thermal processing of plant-derived foods such as coffee beans, nuts, and peppers have demonstrated that heat treatment can induce partial hydrolysis of triacylglycerols and the release of membrane lipids, thereby enhancing the extractability of lipophilic components and promoting the formation of volatile flavor compounds (Tu et al., 2021; Shi et al., 2022; Jiang et al., 2025; Wang et al., 2025). The oil content of flue-cured tobacco leaves is a pivotal physicochemical trait for evaluating the quality of tobacco leaves. Previous studies have shown that, under appropriate moisture conditions, leaves with higher oil content are characterized by darker coloration, a softer and smoother texture, and greater fullness, which collectively contribute to improved aroma quality (Xie et al., 2014). Furthermore, oil in tobacco leaves has been reported to be mainly derived from soft or semi-fluid substances within leaf cells (Gao et al., 2024), these substances include lipids, resins, waxes, and diterpenoids (Chen et al., 2025; Tian et al., 2025). Accumulate or migrate of these substances to the leaf surface during the curing process is determining factor in the oily characteristic of cured leaves (Yan et al., 2007) Petroleum ether extract is frequently utilized as an objective measure (Chen et al., 2013; Bouchnak et al., 2023), as it contains a high concentration of volatile oils, lipids, and resins, and exhibits a strong correlation with oil content (Bouchnak et al., 2023). However, oil accumulation is not simply a reflection of chemical composition; rather, it is the result of a complex metabolic network. Lipid droplets (LDs), the cellular carriers of oil, directly reflect the dynamic balance of triacylglycerol (TAG) synthesis and degradation (Qin et al., 2023; Liang et al., 2025), and play a significant role in determining oil accumulation (Kelly et al., 2013; Chu et al., 2022). The formation of oil content in tobacco leaves has traditionally been categorized within a ternary framework of “variety-cultivation-curing,” with all three factors collectively determining oil expression(Xie et al., 2014). Starch serves as the primary non-structural carbon reservoir in leaves, and its scale directly determines the carbon flux available for lipid synthesis. When starch accumulation decreases, excess carbon flux is significantly redirected toward fatty acid and TAG pathways, while enhanced lipid synthesis conversely inhibits starch accumulation (Vanhercke et al., 2014; Xu and Shanklin, 2016; Yu et al., 2018; Chu et al., 2022; Zhao et al., 2025). Genotypes establish the foundation for carbon allocation and metabolic potential in tobacco leaves. Genome-wide association studies (GWAS) reveal significant differences in initial leaf starch content among tobacco germplasm, with these variations having a clear genetic basis and being jointly regulated by multiple genetic loci (Xu et al., 2022). Within this genetic framework, cultivation management further reshapes the initial chemical and structural background of leaves. Different planting patterns, plastic mulching methods, and fertilization conditions all influence starch accumulation and cell structure, thereby altering the potential carbon supply basis for post-roasting oil formation (Xie et al., 2014). After establishing an initial state jointly determined by genetics and cultivation, roasting—as a critical physiological processing stage—further shapes the final expression of oil content. Tobacco curing is a classical process that promotes senescence and apoptosis (Li et al., 2016; Wu et al., 2020b). Sugars and phospholipids release fatty acids during degradation, which can be re-esterified into TAG and thereby promote oil accumulation (Li et al., 2015; C. Zhang et al., 2020; Cohen et al., 2022). Unlike postharvest-inactivated fruits such as sea buckthorn, whose drying requires external disruption to break the waxy layer (Kaseke et al., 2021; Tan et al., 2022), tobacco leaves maintain cellular activity and antioxidant capacity during the yellowin stage, providing a unique physiological basis for lipid release and translocation. Evidence from coffee roasting shows that heating disrupts the structures of oil bodies and membrane lipids, facilitating lipid migration toward the surface (Wang et al., 2025), Lipidomic analyses further demonstrate that heating strongly activates glycerophospholipid metabolism, marking membrane lipid degradation and remodeling and representing the crucial initiation step of lipid translocation (Wang et al., 2022). However, even under highly standardized production conditions, we observed substantial variation in oil content among cured leaves. This indicates that, beyond the mainstream regulatory framework, additional secondary sources of variation may exist, potentially driven by the intrinsic physiological state or fine-scale metabolic regulation of tobacco leaves; the specific contributing mechanisms remain unclear. Broad-target metabolomics captures biochemical endpoints in organisms and has successfully identified key metabolites in studies of complex quality traits across various crops (Yang et al., 2021; Meng et al., 2022; Shi et al., 2022; Sun et al., 2024). Previous studies have demonstrated significant variations in lipid composition among fresh leaves from different growing regions (Li et al., 2015). To improve the accuracy and specificity of the analysis, this study selected tobacco leaves of the same cultivar and grade that were produced in the same production area under identical cultivation and curing conditions. According to sensory evaluation, the samples were divided into low- and high-oil groups for comparison. This design was intended to control the influence of external factors, including cultivar, environment, agronomic practices, and curing procedures, thereby allowing the analysis to focus on intrinsic differences in oil content determined by the physiological and metabolic characteristics of the tobacco leaves. On this basis, petroleum ether extract content, leaf flexibility scores, color measurements, ultrastructural observations, and non-targeted metabolomic analyses were integrated. Using the tobacco cultivar ‘Yunyan 87’ as the experimental material, shared differential metabolites among samples from different production areas were screened, with the aim of identifying key metabolites stably associated with the oil content trait and analyzing the biological pathways potentially related to these metabolites. This approach provides a basis for exploring the metabolic foundation underlying oil formation in tobacco leaves.

## 2 Materials and methods

### 2.1 Plant growth and sampling

The flue-cured tobacco cultivar Yunyan 87 was cultivated in two distinct regions (**Supplementary Figure 1**): TR (Tongren, Guizhou, China; 107°59′25.32″ E, 27°22′26.99″ N) and WN (Weining, Guizhou, China; 103°99’87.71”E, 26°75’37.86”N), Soil characteristics of the two regions are summarized in **Supplementary Table 1**, and the corresponding climatic parameters are presented in **Supplementary Table 2**. A randomized complete block design was implemented at each site. Each plot covered 121 m² with a planting density of 1.1 m×0.55 m. Three replicates were established per site. Seeds were sown on January 20 and transplanted on April 25. Basal fertilization consisted of 525 kg·ha^-^¹ compound fertilizer (N:P:K = 10:10:25), 450 kg ha^-^¹ fermented oilseed cake, and 375 kg·ha^-^¹ micronutrient fertilizer. Calcium–magnesium phosphate fertilizer was applied simultaneously as part of the basal fertilization program, with the rate determined according to local agronomic recommendations. On the day of transplantation, each plant was watered with 150–200 mL of a water-soluble solution containing 1% compound fertilizer and 0.28% cypermethrin emulsifiable concentrate. Ten days after transplantation, 100–150 mL per plant of a 4% compound fertilizer solution was applied. This fertilization schedule was repeated within 30 days after transplantation, maintaining consistent solution concentrations and application volumes. Plants were topped at the early flowering stage on July 5. Immediately thereafter, leaves at the 4th to 5th positions from the top were labeled to ensure uniform maturity and appearance at harvest. Subsequently, on August 20 at the mature stage, the labeled leaves were harvested. Following the curing process, tobacco leaves were graded as B2F by three experienced tobacco experts based on uniform external appearance. B2F is defined as an intermediate maturity grade according to the Chinese national standard GB 2635-1992, characterized by mature leaves with a moderately loose structure, slightly thick lamina, oil content ranging from present to high, intermediate color, a leaf length of not less than 40 cm, and a damage rate of no greater than 20% The leaves were then selected from both regions. Following a thorough sensory evaluation by experts in the field, the leaves were categorized into two distinct groups, namely high-oil and low-oil, with the objective of ensuring uniformity in leaf position and visual quality among the samples. Each treatment comprised three biological replicates, with each replicate consisting of 20 leaves. A half-leaf sampling approach was adopted, with one half of the leaf used for metabolomic profiling and the other for physicochemical measurements.

### 2.2 Ultrastructural observation

The ultrastructural observations of tobacco leaves were performed according to the method described by Wu et al. (2020a). and the samples were first fixed overnight at 4 °C in 3% glutaraldehyde, followed by sequential treatment with 10% (w/v) picric acid for 2 h and 2% uranyl acetate for another 2 h. Thereafter, the samples were stained with 0.1% (v/v) osmium tetroxide prepared in 150 mM phosphate buffer (pH 7.2). After the completion of fixation and staining, the samples were dehydrated through a graded ethanol series and subsequently infiltrated with EPON812 medium grade resin (Polysciences, Germany). The resin-infiltrated samples were polymerized at 60 °C for 48 h, and ultrathin sections (90 nm) were cut from the polymerized resin blocks using an Ultracut E microtome (Reichert). The sections were collected on formvar-coated grids (EMS, WA), and the ultrastructure of palisade tissues in leaves with different oil contents after curing was finally examined and imaged using a Hitachi H-600 transmission electron microscope (Kyoto, Japan) operating at an accelerating voltage of 75 kV.

### 2.3 Softness value of tobacco leaves

The measurement of tobacco leaf softness value was conducted following the method of Wu et al. (2020a). The leaf softness value was determined by RH-R1000 softness tester (Guangzhou Runhu Instrument Co., Ltd., Guangzhou, China), with rectangular leaf samples placed in the measurement slot. The instrument quantified leaf softness value by recording the combined bending resistance and lateral frictional force, and the results were expressed in millinewtons (mN). For each replicate, samples measuring 5 x 2 cm were collected from the following regions of the leaves: the tip (3rd–4th veins), the middle (6th–7th veins), and the basal (11th–12th veins). The measurements were conducted within a temperature range of 22 ± 1 °C and a relative humidity range of 60 ± 2%.

### 2.4 Leaf color parameters

The leaf color parameters were measured using a CR-10 colorimeter (Konica Minolta Sensing Inc., Tokyo, Japan) on cured tobacco leaves of different oil content groups. Before measurement, the instrument was calibrated with a standard white plate, and each leaf was measured six times at the central lamina area, avoiding the midrib. Color parameters were expressed as the international CIELAB values: luminosity (L*), red-green axis (a*), and yellow-blue axis (b*).Ten tobacco leaves were analyzed for each sample group.

### 2.5 Starch content

The contents of starch was determined following the procedures described by Gong et al. (2023). The chemical composition analyses were performed using a continuous flow analyzer (AA3, Bran+Luebbe GmbH, Germany). Specifically, starch was purified by ultrasonication in an 80% ethanol–saturated NaCl solution and subsequently extracted with 90% DMSO–HCl under heat-assisted ultrasonication. Following the completion of the colorimetric reaction, the quantity of starch was measured using a spectrophotometer.

### 2.6 Petroleum ether extract content

The petroleum ether extract content was determined according to the method described by Sun et al. (2023). A quantity of 15.00 g of finely ground flue-cured tobacco powder was placed in a cellulose extraction thimble and extracted with 100 mL of petroleum ether (boiling range 60–90 °C) using a Soxhlet apparatus. The extraction process was conducted under reflux conditions for a duration of two hours, with the objective of ensuring complete dissolution of lipophilic components. The residue was subsequently re-extracted with an additional 100 mL of petroleum ether for 1.5 hours to improve extraction efficiency. The combined extracts were subjected to filtration, followed by concentration under reduced pressure to remove the solvent. The extracts were then dried to a constant weight. The petroleum ether extract content was calculated as the ratio of extract weight to the initial dry sample weight, and expressed as a percentage (%).

### 2.7 Metabolomic profiling

For solid samples, 0.15 g of homogenized leaf powder was transferred into a 2 mL reinforced tube containing two stainless-steel beads and 1 mL of pre-cooled (−20 °C) methanol–water (7:3, v/v; LC–MS grade, Thermo Fisher Scientific). Samples were homogenized at 50 Hz for 5 min, vortexed three times (30 s each, 10 min intervals), and incubated at 4 °C overnight. The following day, samples were vortexed and centrifuged at 13 000 × g for 10 min at 4 °C. Approximately 800 μL of the supernatant was collected and filtered through a 0.22 μm membrane for LC–MS analysis. For liquid samples, 200 μL of extract was mixed with 600 μL of pre-cooled extraction solvent (methanol: water = 7:3, v/v), incubated at −20 °C for 4 h or overnight, centrifuged at 20 000 × g for 15 min at 4 °C, and filtered through a 0.22 μm membrane. Chromatographic separation was performed on an HSS T3 column (2.1 × 100 mm, 1.8 μm; Waters) maintained at 40 °C. The mobile phases were 0.1% formic acid in water (A) and 0.1% formic acid in acetonitrile (B), with a flow rate of 0.30 mL/min. The gradient program was as follows: 0–2 min, 5% B; 2–22 min, 5–95% B; 22–27 min, 95% B; 27.1–30 min, 5% B. Mass spectrometry was performed on a QTRAP 6500 Plus system (AB Sciex) in multiple reaction monitoring (MRM) mode with an ESI-Turbo ion-spray interface. Source parameters were: 450 °C, spray voltage +5500 V (positive mode)/−4500 V (negative mode), curtain gas 20 psi, and gases I and II at 40 psi. Precursor–product ion pairs, collision energy (CE), decluttering potential (DP), and retention times were recorded for targeted metabolites. Ion peak extraction and metabolite identification/quantification were conducted using Skyline (v.21.1.0.146) with Mass Tolerance set to 0.6 Da and Mass Range 50–1500 Da, referenced against the BGI-Wide Target-Library. The raw LC–MS data were processed in metaX: Features with more than 50% missing values in QC samples and more than 80% missing values in experimental samples were removed to minimize unreliable measurements. Coefficients of variation (CV) were calculated for the remaining features using QC samples, and features exhibiting a CV >30% were excluded in order to ensure analytical reproducibility and signal stability. The remaining missing values were imputed using the k-nearest neighbor (KNN) algorithm. The data underwent a process of normalization, employing probabilistic quotient normalization (PQN) as the primary method. This was followed by a batch effect correction procedure that utilized QC-based robust LOESS signal correction (QC-RLSC). The quality control (QC) assessment was conducted by evaluating the CV distributions and performing principal component analysis (PCA) on the QC samples. This confirmed instrument stability and the suitability of the retained feature set for downstream statistical analysis and metabolic pathway interpretation. In order to monitor system stability, pooled QC samples were inserted at a ratio of one QC per three analytical samples. Metabolite data were log_2_-transformed and Pareto-scaled prior to principal component analysis (PCA) to evaluate sample distribution, group separation, and QC clustering, thereby assessing analytical reproducibility and batch consistency. Metabolites were subsequently classified and functionally annotated using the Kyoto Encyclopedia of Genes and Genomes (KEGG) database to facilitate interpretation of biological functions and identification of potential metabolic pathways. Metabolite classification and functional annotation were based on the KEGG (Kyoto Encyclopedia of Genes and Genomes) databases to interpret biological functions and potential metabolic pathways.

### 2.8 Data analysis

All experimental data were subjected to statistical analysis using Excel and R-Studio, and the results are expressed as the mean ± standard deviation. Graphs and visualizations were generated using Origin and R software. Metabolomic data were analyzed and visualized in the R environment. Multiple-group comparisons were performed using a one-way analysis of variance (ANOVA) followed by a Duncan’s *post hoc* test. Metabolite-pathway associations were retrieved from the KEGG database via the KEGGREST package, and pathway enrichment was assessed using a hypergeometric test with Benjamini–Hochberg correction. The results of the enrichment process were presented in the form of RichFactor-based dot plots, for which the ggplot2 and scales packages were utilized.

## 3 Results and discussions

### 3.1 Variations in physical characteristics of cured tobacco leaves with region and oil-content grouping

**Figure 1:**
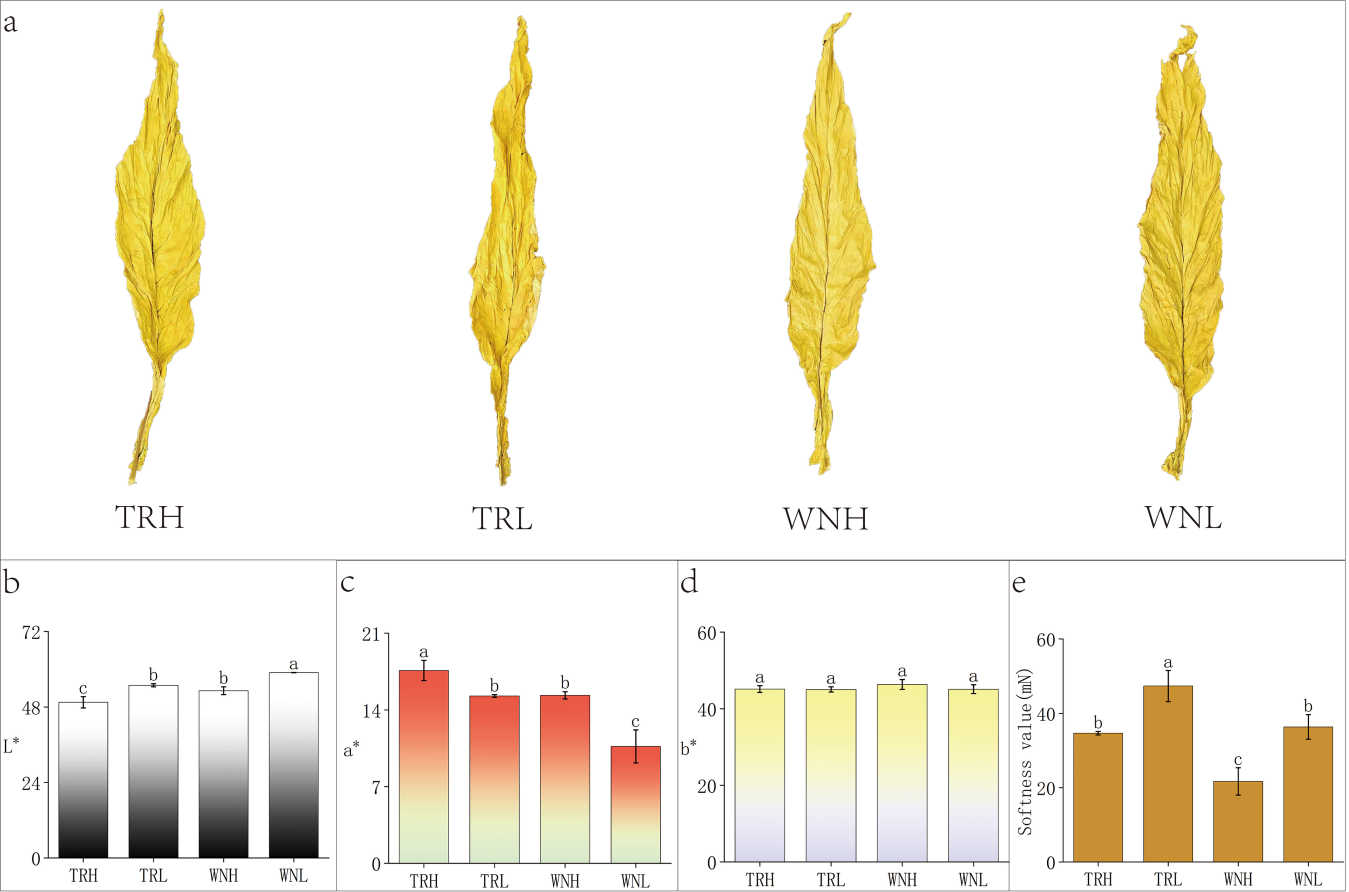
Morphology, color parameters, and softness of tobacco leaves from different regions and oil-content groups. **(A)** Morphology of High- and Low-Oil Tobacco Leaves from Two Regions. From left to right: TRH (high-oil, Tongren), TRL (low-oil, Tongren), WNH (high-oil, Weining), WNL (low-oil, Weining). **(B)** Luminosity (L*); **(C)** Red-Green Axis (a*); **(D)** Yellow-Blue Axis (b*); **(E)** Softness value (mN; lower values indicate greater softness). Data are shown as mean ± SD (n = 3 biological replicates). Different lowercase letters indicate significant differences at p < 0.05 (Duncan’s multiple range test).

The morphological disparities between high- and low-oil tobacco leaves from the WN and TR regions are illustrated in **Figure 1A**. It was observed that high-oil leaves from both regions exhibited a softer texture, greater ease of flat enation, and a glossy, oily appearance. In contrast, low-oil leaves exhibited greater density and softness value. They demonstrated minimal spreading when subjected to compression, and exhibited a paucity of surface gloss. The color characteristics of cured tobacco leaves in WN and TR exhibited consistent differences between oil content groups and across regions (**Figures 1B–D**). High-oil leaves exhibit a darker appearance (Xie et al., 2014). Within the same region, high-oil leaves exhibited significantly lower L* values and higher a* values compared to low-oil leaves, whereas b* values did not differ significantly between treatments. Cross-regional comparisons revealed that, at equivalent oil content levels, leaves from WN generally exhibited higher L* values and lower a* values in comparison to those from TR. Lower softness value values are indicative of greater leaf softness value (**Figure 1E**), and overall, leaves from WN exhibited the lowest softness values. Within the same region, high-oil leaves exhibited significantly lower softness values (21.67 mN) in comparison to low-oil leaves (36.33 mN).

### 3.2 Ultrastructural observation of lipid droplets in palisade mesophyll cells

**Figure 2:**
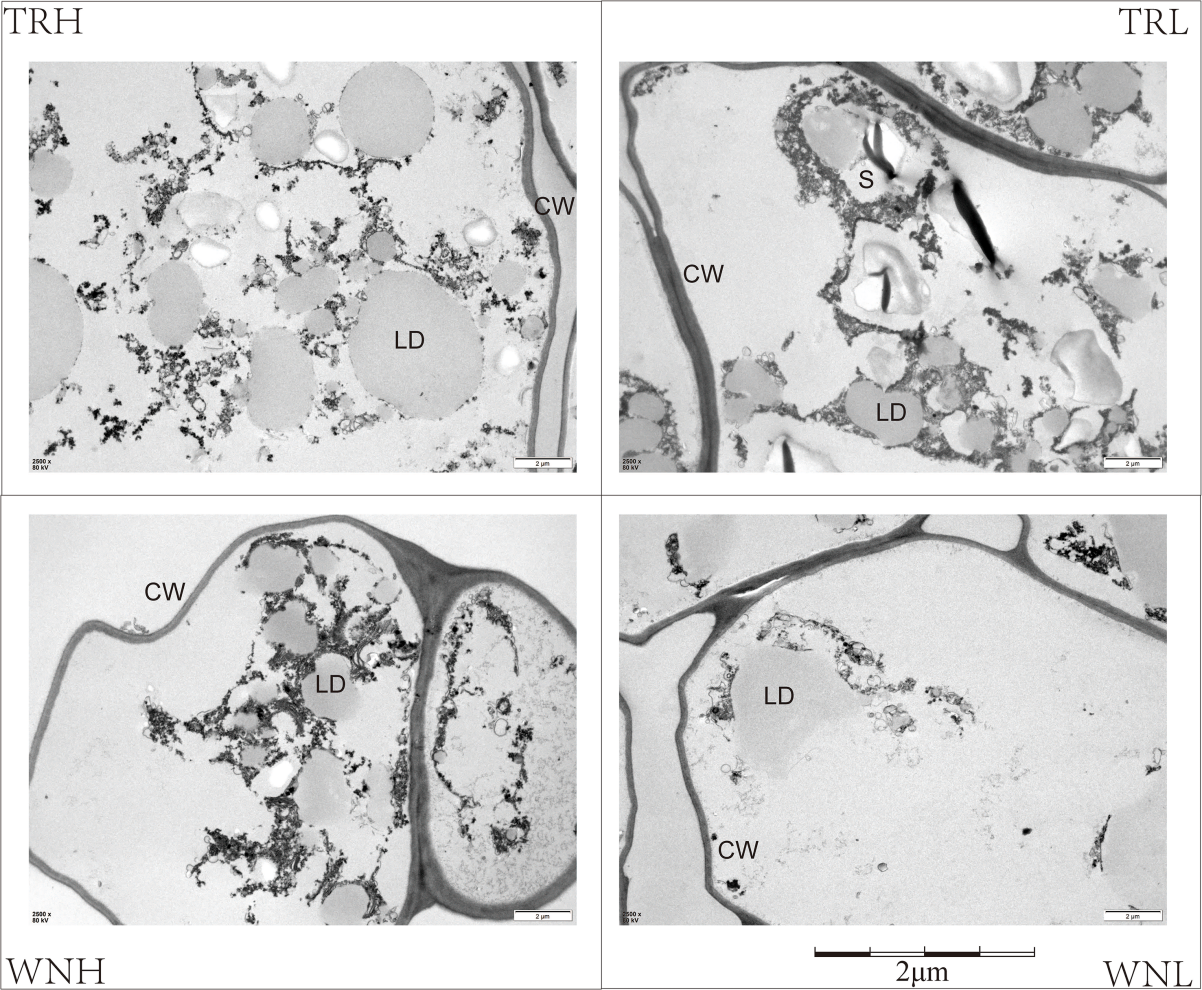
Ultrastructural observation of palisade cells. Scale bar = 2 μm. LD, lipid droplet; S, starch granule; CW, cell wall.

As illustrated in **Figure 2**, the cellular ultrastructural characteristics of high- and low-oil tobacco leaves from diverse regions are presented. As demonstrated in previous studies, there is a demonstrable correlation between the number, size, and spatial distribution of LDs and oil accumulation (Vanhercke et al., 2017; Qin et al., 2023; Liang et al., 2025), the content of LD was found to be highest in TRH, followed by WNH and TRL, and lowest in WNL. A comparison of regional data sets revealed that the LD content in the TR region generally exceeded that observed in the WN region. Within the same region, high-oil leaves consistently contained greater numbers of lipid droplets than low-oil leaves. It is noteworthy that pronounced starch granule residues were observed in TRL leaves. The curing process has been shown to enhance starch degradation, resulting in higher oiliness and aroma intensity in high-oil leaves (Fan et al., 2019a).

### 3.3 Oil and starch content variations in regional and oil-content grouping

**Figure 3:**
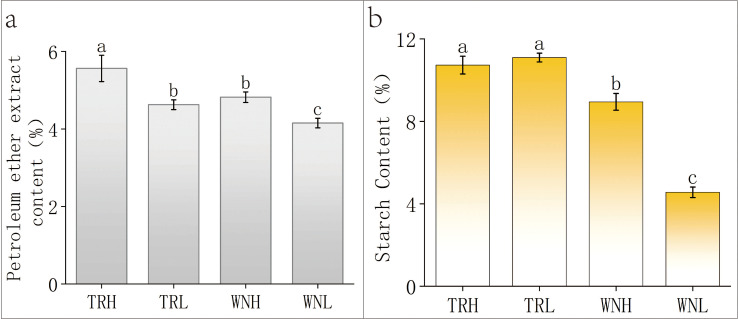
Petroleum ether extract and starch contents of tobacco leaves from different regions and oil-content groups. **(A)** Petroleum ether extract content (%); **(B)** Starch content (%). Data are shown as mean ± SD (n = 3 biological replicates). Different lowercase letters indicate significant differences at p < 0.05 (Duncan’s multiple range test).

The content of petroleum ether extract in tobacco leaves has been shown to serve as an indicator of oil content (Yan et al., 2007). The petroleum ether extract content (**Figure 3A**) was significantly higher in high-oil samples than in low-oil samples within the same region. Based on the quantitative distribution of petroleum ether extract content across the two regions over the past two years (**Supplementary Figures 2**, **S3**), the high- and low-oil categories defined in this study were consistent with the natural separation observed in the dataset. This was accompanied by a greater abundance and larger size of LDs. The starch content (**Figure 3B**) exhibited a significant variation among the oil content groups in WN, with high-oil leaves (8.95%) demonstrating a reduced starch content in comparison to low-oil leaves (4.56%). Conversely, in the TR region, starch levels were comparable between high-oil (10.73%) and low-oil (11.09%) leaves, with no significant difference While previous studies have generally identified a positive correlation between tobacco leaf oil content and starch content, consistent with the observational data from the WN region, the atypical absence of significant variation in total starch content in TR tobacco leaves stems from the region’s inherently high starch content (Li et al., 2025 The Impact of Major Meteorological Factors in Tobacco Growing Areas on Key Chemical Constituents of Tobacco Leaves). It is the combination of this inherent regional trait and the incomplete starch conversion in low-oil TR leaves (where residual starch exists in granular form) that results in the atypical characteristic of comparable starch levels between high- and low-oil leaves in the TR region, a fact evidenced by the presence of residual starch granules in low-oil TR samples after curing While previous studies have generally identified a positive correlation between tobacco leaf oil content and starch content, consistent with the observational data from the WN region, the atypical absence of significant variation in total starch content in TR tobacco leaves stems from the region’s inherently high starch content (Li et al., 2025). It is the combination of this inherent regional trait and the incomplete starch conversion in low-oil TR leaves (where residual starch exists in granular form) that results in the atypical characteristic of comparable starch levels between high- and low-oil leaves in the TR region, a fact evidenced by the presence of residual starch granules in low-oil TR samples after curing. In WN, high-oil samples exhibited significantly higher starch content in comparison to low-oil samples. Given that all leaves were harvested at comparable maturity stages, this variation is likely associated with differences in starch degradation and conversion rates. It has been hypothesized by researchers in the past that the process of oil formation in leaves is associated with the reallocation of carbon between starch and lipid pools. This is in addition to the dynamic equilibrium between the synthesis and degradation of TAG (Andrianov et al., 2010; Zhao et al., 2025). Tobacco leaves manifest a “non-transient starch accumulation” pattern during natural development. In this pattern, a proportion of the carbon stored in starch is reallocated to lipid synthesis under specific conditions. This process has the capacity to influence oil content levels (Sanjaya et al., 2011). Consequently, the efficiency of starch degradation and carbon flux towards lipid biosynthesis may represent a pivotal mechanism determining leaf oil potential. Correlation analyses from previous studies have reported a positive relationship between post-cured oil content and starch levels in tobacco leaves (Xie et al., 2015; Zuo et al., 2023), consistent with the region-specific patterns observed in TR and WN.

### 3.4 Sample quality control and overview of metabolites

**Figure 4:**
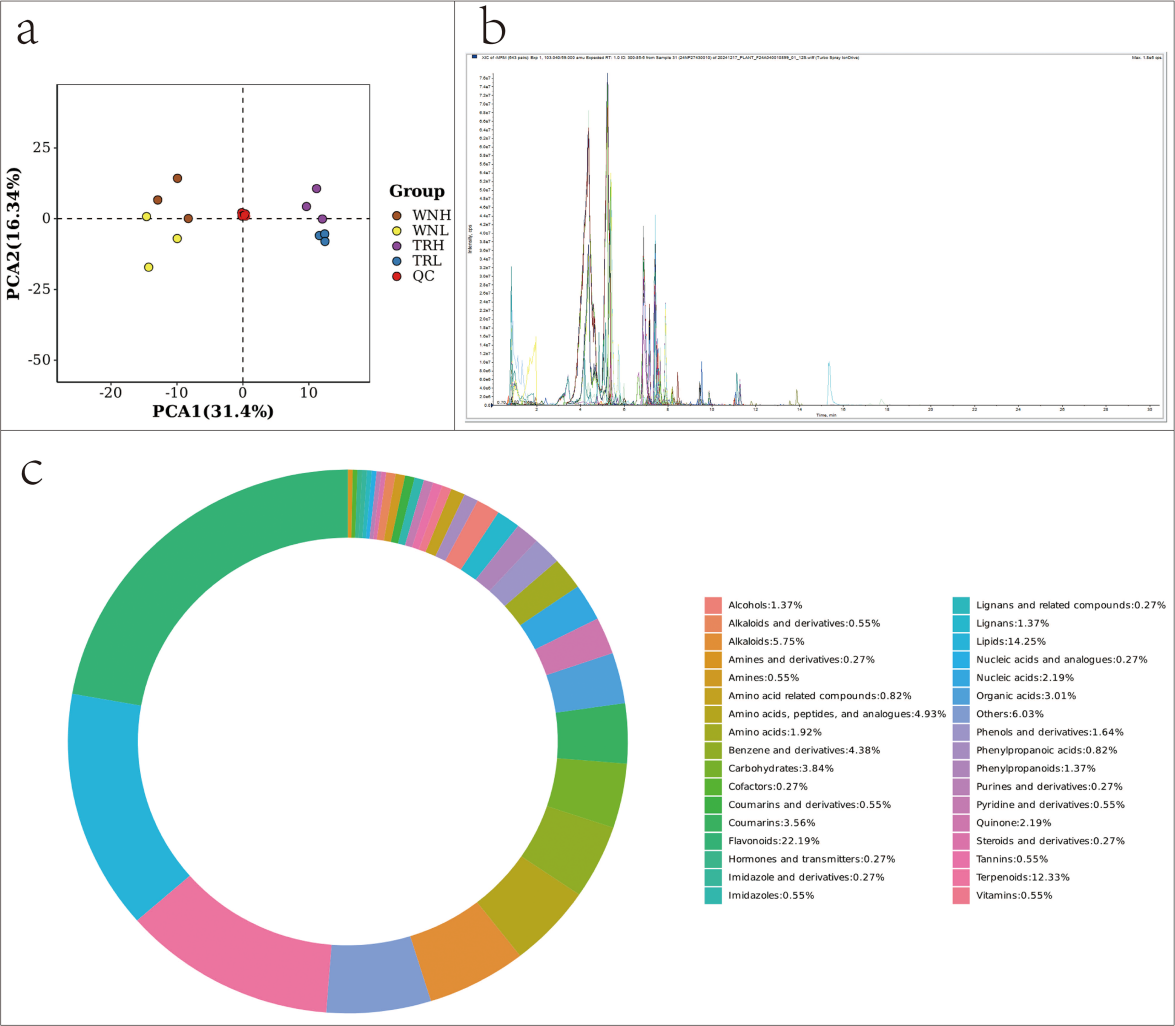
Overview of metabolomic profiling in cured tobacco leaves. **(A)** PCA of all samples; **(B)** Extracted ion chromatograms (XICs) of representative metabolites; **(C)** Distribution of identified metabolite classes;.

As illustrated in **Figure 4A**, the PCA of the cured tobacco leaf samples is demonstrated. The first principal component (PC1, 31.4%) primarily separates samples from different regions, while the second principal component (PC2, 16.34%) principally reflects differences in oil content, thus indicating distinct groupings in the metabolite profiles. In order to assess the stability and reliability of metabolite detection, **Figure 4B** presents the extracted ion chromatograms (XICs) of representative metabolites, showing clear peak shapes, consistent retention times, and peak areas suitable for quantification. As illustrated in **Figures 4C**, the identified metabolite classes demonstrate a predominance of flavonoids (22.19%), lipids (14.25%), and terpenoids (12.33%). The remaining metabolites encompass alkaloids, amino acids and derivatives, organic acids, carbohydrates, phenylpropanoids, vitamins, and hormones.

### 3.5 Identification and classification of differential metabolites

**Figure 5:**
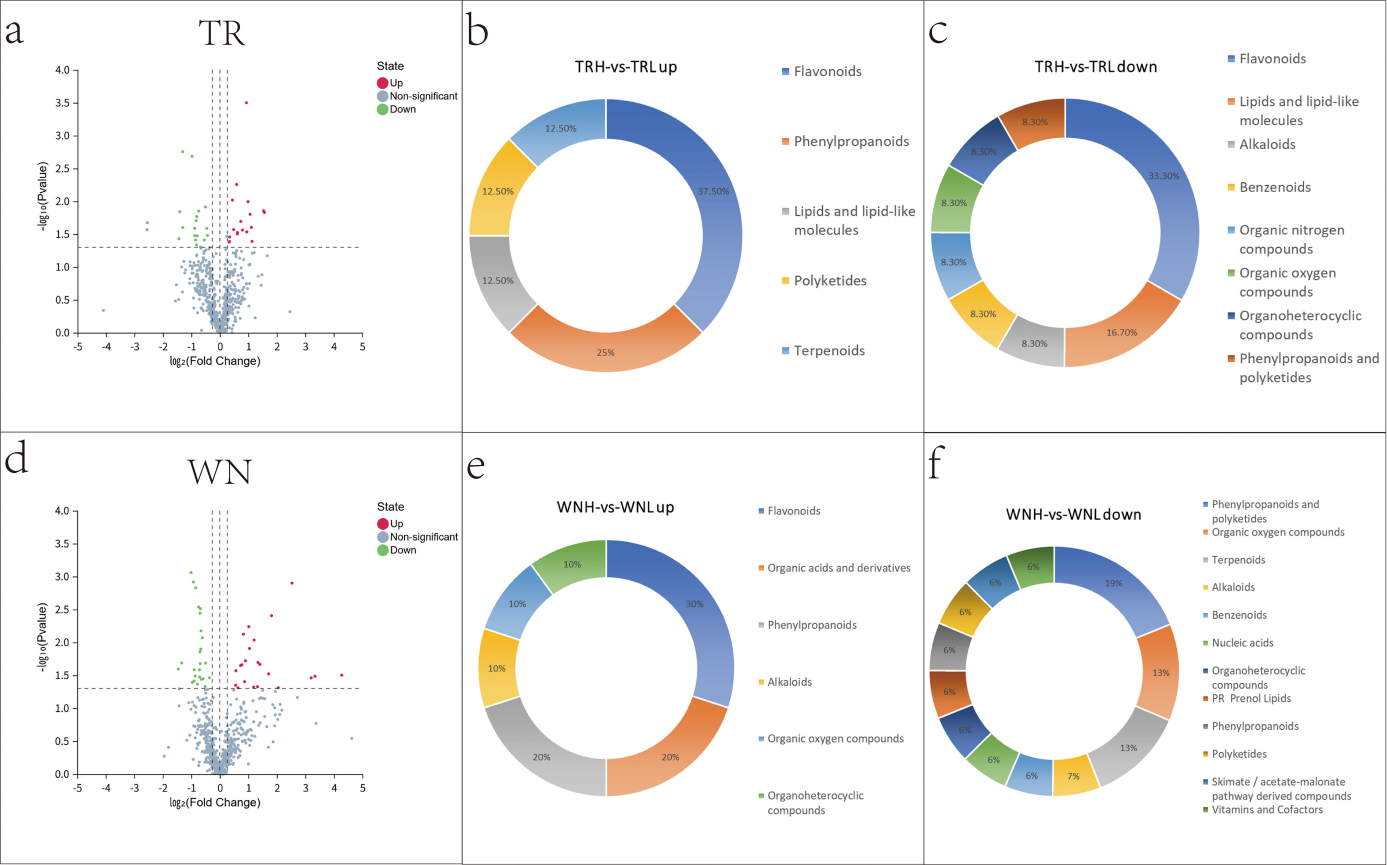
Profiling of differential metabolites from high- vs. low-oil comparisons. **(A)** TR volcano plot; **(B)** TR up-regulated classes; **(C)** TR down-regulated classes; **(D)** WN volcano plot; **(E)** WN up-regulated classes; **(F)** WN down-regulated classes.

The selection of differential metabolites was based on the following criteria: FC ≥ 1.2 or ≤ 0.8, VIP ≥ 1 and p-value ≤ 0.05. The comparison between the high- and low-oil groups within each region (**Figures 5A, D**) revealed 38 differential metabolites in TR (TRH vs TRL), including 18 that were found to be upregulated and 20 that were found to be downregulated, and 47 differential metabolites in WN (WNH vs WNL), including 22 that were found to be upregulated and 25 that were found to be downregulated. For a more comprehensive overview refer to **Supplementary Table 4**, which provides detailed information on the subject. Further analysis of differential metabolites in TR (**Figures 5B, C**) demonstrates that the upregulated metabolites are primarily comprised of triglycerides, phospholipids, and fatty acid derivatives, while the downregulated metabolites are predominantly enriched in polyphenols and phenylpropanoids. In contrast, in WN (**Figures 5E, F**), the predominant upregulated metabolites comprise flavonoids, coumarins and sesquiterpene lactones, which are classified as polar or semi-polar compounds. Conversely, downregulated metabolites are predominantly concentrated in organic acids, sugar alcohols and certain fatty acid derivatives. These observations are consistent with the measurements of petroleum ether extract and the distributions of lipid droplets, indicating that TRH exhibit greater lipid synthesis and storage capacity, whereas WN samples show enhanced activation of aromatic secondary metabolites.

Pearson correlation analysis was performed between region-specific differential metabolites and petroleum ether extract content, and the results are presented in the **Supplementary Table 5**. In both regions, multiple flavonoids, phenylpropanoids, and terpenoid metabolites were significantly correlated with petroleum ether extract content, constituting a common biochemical basis underlying leaf oiliness and softness. In high-oil tobacco leaves from Weining, free and esterified lipophilic metabolites exhibited significantly higher abundances, including the sesquiterpene ester farnesyl acetate, the isoquinoline alkaloid boldine, and the flavonol myricetin, all of which are lipophilic compounds. The abundances of robinin and 3-chloro-L-tyrosine were positively correlated with petroleum ether extract content. Robinin, a potent antioxidant flavonoid, has been reported to enhance drought tolerance in chrysanthemum under exogenous application (Elansary et al., 2020). 3-Chloro-L-tyrosine is widely recognized as a biomarker of oxidative damage in biological systems, although evidence in plants remains limited. In low-oil tobacco leaves, the abundances of the water-soluble bitter glycoside gentiopicrin and the oligosaccharides raffinose and stachyose were negatively correlated with petroleum ether extract content. Gentiopicrin is a secoiridoid glycoside derived from monoterpenoids, and its biosynthesis shares common precursors with the monoterpenoid biosynthesis pathway. When metabolic flux is preferentially channeled toward lipophilic terpenoids, volatile oils, pigments, and other petroleum ether-extractable components, flux toward polar glycosides such as secoiridoid glycosides is correspondingly reduced, resulting in a significant negative correlation (Van Der Sluis and Labadie, 1981). Raffinose and stachyose belong to the raffinose family oligosaccharides (RFOs). Their primary physiological role is to participate in stress responses, enhancing tolerance to dehydration, high temperature, and oxidative stress through osmotic adjustment, membrane stabilization, and antioxidant activity (Elango et al., 2022). The biosynthesis of RFOs consumes soluble sugars derived from starch degradation, thereby generating competition for precursor substrates with lipid biosynthesis. In high-oil tobacco leaves from Tongren, glycosylated metabolites were positively correlated with petroleum ether extract content, including the isoflavone glycoside ononin, the diterpene glycoside steviol-19-O-glucoside, the triterpenoid saponin ginsenoside Rg2, and the coumarin glycoside nodakenin. These compounds are stored in inactive glycosylated forms and release their aglycones through hydrolysis during curing, aging, or combustion, thereby serving as aroma precursors (Cai et al., 2013; Li et al., 2022). In contrast, vitexin and isovitexin were negatively correlated with petroleum ether extract content. As C-glycosyl flavonoids, they exhibit high structural stability (Lam et al., 2023) and are less susceptible to hydrolysis or degradation during curing. Their accumulation therefore more likely reflects sustained aromatic secondary metabolism prior to or during curing rather than direct involvement in oil formation. Similarly, the negative correlation between 4-methyl-octanoic acid and petroleum ether extract content suggests compositional differences in lipid metabolic profiles in low-oil samples. However, as this analysis was conducted using post-curing samples, it remains unclear whether this association reflects reduced lipid deposition or lipid remodeling during the curing process.

### 3.6 Divergent metabolic drivers underlie oil content variation in TR and WN

**Figure 6:**
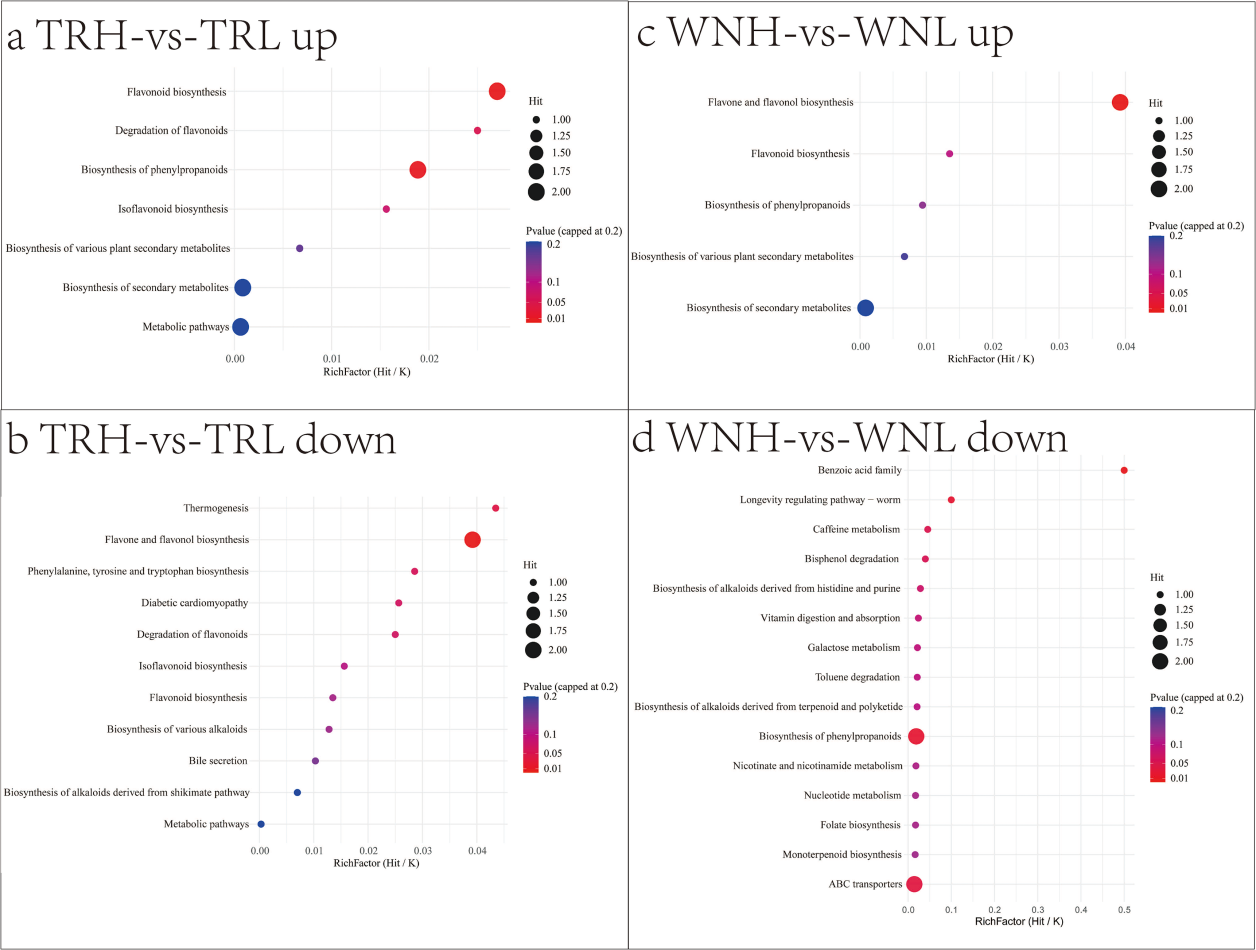
KEGG enrichment analysis of up- and down-regulated metabolites from high- vs. low-oil comparisons in TR and WN regions. **(A)** TR-up; **(B)** TR-down; **(C)** WN-up; **(D)** WN-down.

Enrichment analysis (**Figures 6A–D**) revealed markedly distinct metabolic states between high-oil tobacco in the TR and WN regions. KEGG enrichment analysis was performed using the hypergeometric test (Over-Representation Analysis, ORA), with all KEGG compounds designated as the background set, and p-values were adjusted using the Benjamini–Hochberg method. This study demonstrates that although TR and WN flue-cured tobacco both exhibit a high-oil phenotype, their metabolic foundations differ substantially. Differential metabolites in both regions were enriched in the flavonoid biosynthesis and phenylpropanoid biosynthesis pathways, indicating that these two pathways constitute a common basis underlying regional differences in petroleum ether extract content. Notably, metabolic resource allocation in WN tobacco leaves was strongly directed toward terminal modification and accumulation of flavonoids. Significantly upregulated metabolites were primarily enriched in the flavone and flavonol biosynthesis pathway and consisted predominantly of glycosylated flavonol derivatives, including robinin and apigenin-7-O-β-D-glucoside. This pattern indicates activation of late-stage modification steps such as glycosylation during flavonoid metabolism in WN leaves. Glycosylation is generally regarded as a hallmark of metabolic inactivation in plant cells (Sudheeran et al., 2020). This suggests that WN tobacco preferentially channels flavonoid metabolic flux toward the accumulation of stable end products rather than maintaining dynamic turnover. This focused metabolic strategy was accompanied by a pronounced trade-off. Phenylpropanoid metabolism occupies a central hub in plant secondary metabolism, and its precursor phenylalanine also serves as a shared substrate for protein biosynthesis and multiple volatile compounds, including benzoic acid and phenolic acids (Vogt, 2010). In WN tobacco leaves, downstream phenylpropanoid biosynthesis and flavonoid biosynthesis pathways that were positively correlated with petroleum ether extract content were significantly activated. In contrast, several competitive branches derived from the same shikimate pathway exhibited downregulated trends. These included monoterpenoid biosynthesis derived from isopentenyl pyrophosphate, in which gentiopicrin was downregulated; benzoic acid family metabolism directly derived from phenylalanine, in which 4-hydroxybenzoic acid was reduced; and purine and caffeine metabolism, in which guanosine and theophylline were decreased, respectively. Both purine and caffeine metabolism consume phosphoribosyl pyrophosphate, further indicating competitive allocation of metabolic precursors. In contrast to WN, TR tobacco leaves displayed extensive reprogramming of flavonoid-related metabolism rather than a unidirectional accumulation pattern. The dataset included significantly upregulated metabolites in the flavonoid biosynthesis pathway, such as naringenin 7-O-glucoside, as well as significantly downregulated metabolites in the flavone and flavonol biosynthesis pathway, including isovitexin and vitexin. Within the isoflavonoid biosynthesis pathway, ononin was significantly upregulated, whereas genistin showed a decreasing trend. Differential regulation among distinct branches of the flavonoid superfamily indicates that flavonoid metabolic flux in high-oil TR tobacco remains in a dynamic turnover state rather than being directed solely toward terminal product accumulation. Several terpenoid metabolites, including ginsenoside Rg2, decursinol, and dihydrotanshinone I, were significantly upregulated, whereas L-menthyl lactate in the monoterpenoid biosynthesis pathway was significantly downregulated. These results suggest overall activation of terpenoid metabolism accompanied by branch-specific regulatory divergence. Concurrently, alkaloids derived from the shikimate pathway and multiple alkaloids not assigned to defined pathways exhibited complex fluctuations. This multi-pathway, multidirectional, and multinodal metabolic reorganization constitutes the chemical basis of the honey-sweet aroma type associated with high-oil TR tobacco leaves. At the phenylpropanoid and flavonoid hub, TR tobacco did not channel all metabolic flux into a single storage form but instead maintained a balanced distribution among flavonoid biosynthesis, isoflavonoid biosynthesis, and flavone and flavonol biosynthesis.

The markedly divergent metabolic architectures observed between high-oil TR and WN tobacco, particularly the contrasting strategies within flavonoid metabolism, raise a central question: do these differences in secondary metabolic phenotypes reflect distinct underlying mechanisms of lipid accumulation? The present non-targeted metabolomic analysis did not reveal enrichment of the triacylglycerol pathway, indicating that enhanced triacylglycerol synthesis is not a universal driver of high-oil phenotypes in the two regions. However, triacylglycerol accumulation in leaves is governed by the dynamic balance between synthesis and degradation. Adequate carbon supply provides substrates for triacylglycerol biosynthesis, consistent with metabolic engineering studies demonstrating that redirecting carbon flux toward lipid synthesis can markedly increase triacylglycerol accumulation (Sanjaya et al., 2011; Kong et al., 2013; Zhou et al., 2020). Secondly, TAG levels are not solely determined by synthesis rates but are also strongly constrained by degradation and turnover processes (Kong et al., 2013; Vishwakarma et al., 2017; Huang, 2018). Therefore, the increased lipid droplet abundance and petroleum ether extract content observed in high-oil samples from both regions may result from distinct regulatory mechanisms, including enhanced synthesis, reduced degradation, or differential carbon allocation within primary metabolism. Environmental factors such as temperature and water availability have been shown to regulate lipid accumulation in tobacco leaves by maintaining higher activities of triacylglycerol biosynthetic enzymes and promoting carbon flux toward lipid biosynthesis (Nam et al., 2022.; Zhang et al., 2025; Lu et al., 2020; Wei et al., 2024). These findings suggest that environmental conditions may contribute to the observed lipid phenotypes by sustaining elevated basal enzymatic activity and redirecting metabolic flux (Andrianov et al., 2010; Fan et al., 2019b). In contrast, the oil-associated phenotype identified in the present study is characterized primarily by pronounced activation of aromatic secondary metabolism, as evidenced by substantial alterations in flavonoids, oxygenated volatiles, and sesquiterpenoid profiles. This metabolic configuration indicates a greater reliance on secondary metabolic networks, particularly the phenylpropanoid and flavonoid pathways. Discriminating among these potential mechanisms will require further investigation through targeted lipidomics, metabolic flux analysis, and expression profiling of key lipid metabolism genes.

### 3.7 Identification of oil content-associated metabolites conserved across ecological regions

**Figure 7:**
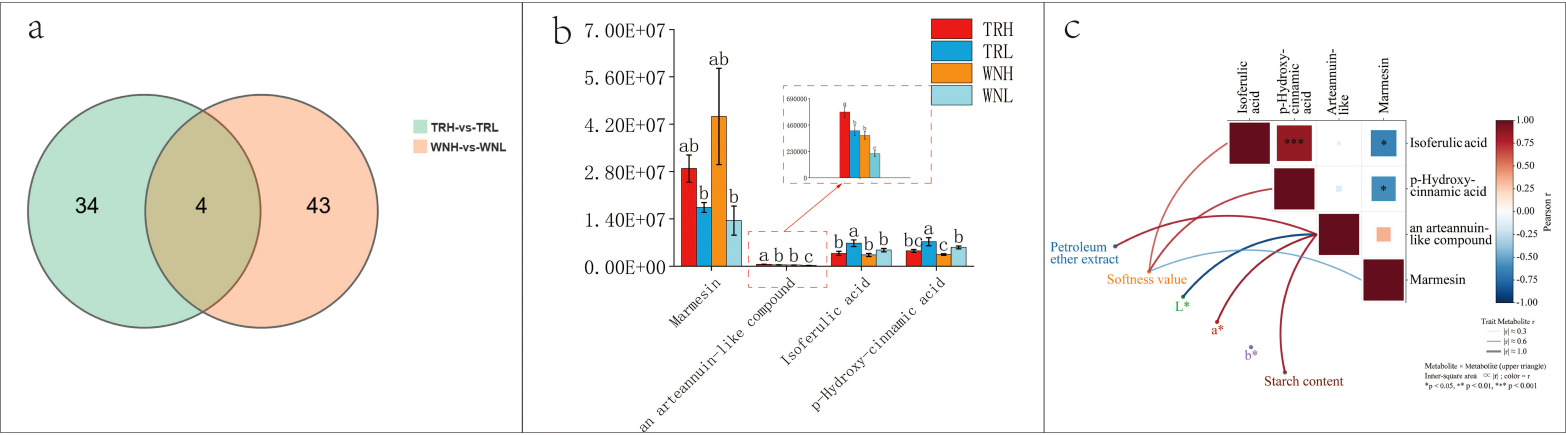
Analysis of metabolites common to high- vs. low-oil comparisons in both regions. **(A)** Venn diagram; **(B)** Metabolite abundance; **(C)** Pearson correlation analysis.

Four metabolites—namely, marmesin, arteannuin, isoferulic acid, and p-hydroxy-cinnamic acid—were identified as common to both regions by Venn analysis (**Figure 7A**), suggesting a stable link to the oil content phenotype. The abundance of the four shared differential metabolites are displayed in **Figures 7B**, and their correlations with color parameters, petroleum ether extract content, starch content, and leaf softness value are presented in **Figure 7C**. The content of arteannuin has been found to be positively correlated with petroleum ether extract (r = 0.82, p = 0.001), starch content (r = 0.838, p = 0.001), and a* (r = 0.86, p < 0.001). In addition, a negative correlation has been observed between arteannuin and L* (r = −0.91, p < 0.001). The softness value exhibited a negative correlation with marmesin (r = −0.55, p = 0.04) and a positive correlation with isoferulic acid (r = 0.61, p = 0.02) and p-hydroxy-cinnamic acid (r = 0.58, p = 0.03). These findings suggest that these metabolites can serve as reference indicators for oil content. It is evident from the correlations that the four shared metabolites can be regarded as potential biomarkers associated with the oil content phenotype. These metabolites may serve to trace oil-related metabolic networks across regions or cultivars. isoferulic acid and p-hydroxy-cinnamic acid are phenylalanine-derived metabolites that participate in the phenylalanine to cinnamic acid and downstream phenylpropanoid pathways. The accumulation of lignin and related phenylpropanoid products has been shown to alter the aromatic composition and mechanical strength of the cell wall, thereby affecting leaf rigidity and softness (Boerjan et al., 2003; Vogt, 2010; Polo et al., 2020). Isoferulic acid, a branch product of the phenylpropanoid pathway, has been demonstrated to be associated with antioxidant activity (Shen et al., 2019; Pang et al., 2018) and is significantly downregulated during fruit ripening (Tallapally et al., 2020). p-Hydroxy-cinnamic acid is an important structural component of lignin, and its abundance is closely linked to lignin biosynthesis, as this metabolite can be released through high-temperature hydrolysis of lignin (Zhuang et al., 2012). In the present study, marmesin content was significantly higher in high-oil tobacco leaves than in low-oil leaves. Marmesin is a lipophilic phenylpropanoid derivative belonging to the coumarin class and is known to participate in antioxidant activity and stress responses (Afek et al., 2002). Although no direct evidence currently links marmesin to tobacco leaf oil content or leaf softness, we speculate that this association may be related to subcellular localization, given that the biosynthetic pathway of marmesin is anchored in the endoplasmic reticulum (Villard et al., 2021), which is also the primary site of lipid synthesis. Enhanced endoplasmic reticulum function may therefore promote the coordinated accumulation of lipids and phenylpropanoid branch metabolites (Wagner & Hrazdina, 1984). Among the shared differential metabolites, one feature annotated as arteannuin (a sesquiterpene lactone) demonstrated a significant association with the oil-related phenotype. However, it is noteworthy that the biosynthesis of artemisinin and related sesquiterpene lactones is highly specific to *Artemisia annua* and related species. It is evident that key enzymes, including ADS, CYP71AV1 and DBR2, are expressed at elevated levels in *A. annua*, thus forming a specialized biosynthetic pathway. In contrast, common tobacco (*Nicotiana tabacum*) is devoid of a complete artemisinin biosynthetic gene cluster (Ikram & Simonsen, 2017). Consequently, it is probable that this annotation is a false positive result based on spectral similarity. Consequently, this feature is tentatively interpreted as an “an arteannuin-like compound”, whose precise chemical nature necessitates further validation through the utilization of authentic standards and high-resolution mass spectrometry. In the domain of metabolomics research, there is a prevailing consensus on the necessity of validating metabolite annotations through a variety of strategies. These strategies encompass the comparison of metabolite annotations with authentic standards, the utilization of high-resolution MS/MS spectra, the consideration of retention time, and the evaluation of ion mobility. The implementation of these strategies is believed to enhance the reliability of metabolite annotations (Xiao et al., 2012), These approaches can serve as a point of reference for future studies in this field. If the annotation is found to be authentic, it may imply that tobacco, under specific ecological conditions, possesses an unusual capacity for sesquiterpene biosynthesis, suggesting the presence of novel terpenoid modification pathways or possible microbial co-metabolism. Alternatively, if it represents a false positive, the signal may still correspond to a structurally similar, similarly oxidized, or functionally analogous terpenoid derivative in tobacco, reflecting potential oxidative terpenoid activity during oil accumulation. Consequently, this signal should not be disregarded outright but regarded as a valuable metabolic indicator that may reveal potential coupling between lipid metabolism and terpenoid biosynthetic pathways. Subsequent studies may employ authentic standards, high-resolution MS/MS spectra, retention time, and ion mobility measurements to further clarify its chemical identity and biological significance.

## 4 Conclusions

The present study employed a cross-regional widely-targeted metabolomic analysis to characterize the oil content phenotype and metabolic profiles of flue-cured tobacco leaves. High-oil leaves contained a greater number of lipid droplets, exhibited higher flexibility (as indicated by significantly lower stiffness values), and had higher petroleum ether extract content than low-oil leaves across both regions. Pronounced starch granule residues were observed in low-oil leaves from TR, whereas WN leaves did not exhibit this feature. A total of 38 differential metabolites and 47 differential metabolites were identified in TR and WN, respectively. In TR, upregulated metabolites mainly included triacylglycerols, phospholipids, and fatty acid derivatives, while downregulated metabolites were enriched in polyphenols and phenylpropanoids. In WN, differentially abundant metabolites comprised flavonoids, coumarins, and sesquiterpene lactones, whereas reduced levels were observed in organic acids, sugar alcohols, and certain fatty acid derivatives. Four metabolites—marmesin, an arteannuin-like compound, isoferulic acid, and p-hydroxy-cinnamic acid—were consistently different between high- and low-oil groups in both regions. The arteannuin-like compound showed a positive correlation with petroleum ether extract content and a* but a negative correlation with L*. Conversely, stiffness was negatively correlated with marmesin and positively correlated with isoferulic acid and p-hydroxy-cinnamic acid. These shared metabolites may serve as metabolic indicators for oil content regulation and reference candidates for cross-regional validation.

## Data Availability

The original contributions presented in the study are included in the article/**Supplementary Material**. Further inquiries can be directed to the corresponding authors.
