# Supplementary material for: Regional metabolomic profiling reveals lipid metabolic signatures associated with oil content in flue-cured tobacco
Source: Front Plant Sci. 2026 Mar 16;17:1734410. doi: 10.3389/fpls.2026.1734410 (PMC13033684; doi:10.3389/fpls.2026.1734410)
Supplement: Supplementary file 1 [file DataSheet1.docx]

Appendix


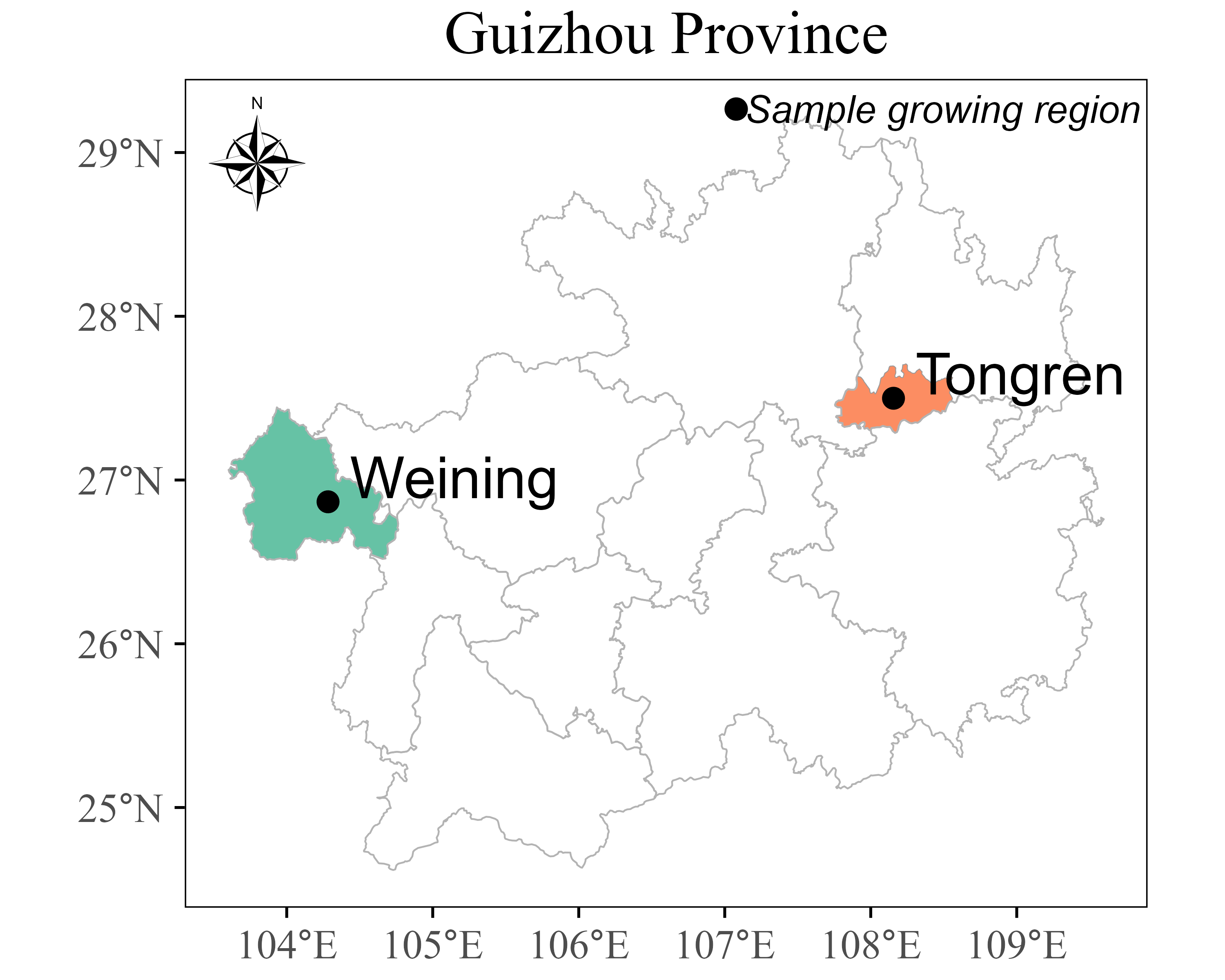


Figure S1 Information on Sampling Points

Table S1 Soil physical and chemical properties of sampling sites in Weining and Tongren

| **Parameter** | **Weining (Surface)** | **Weining (Subsurface)** | **Tongren (Surface)** | **Tongren (Subsurface)** | **Unit** |
| --- | --- | --- | --- | --- | --- |
| Soil type | Luvisol | | Luvisol | / | / |
| Soil texture | Fine | | Fine | / | / |
| Gravel content | 19.0 | 28.0 | 4.0 | 6.0 | % |
| Sand content | 31.0 | 27.0 | 27.0 | 21.0 | % |
| Silt content | 22.0 | 20.0 | 27.0 | 25.0 | % |
| Clay content | 47.0 | 53.0 | 46.0 | 54.0 | % |
| Bulk density | 1.26 | 1.24 | 1.26 | 1.22 | kg/dm³ |
| Organic carbon | 1.2 | 0.59 | 1.2 | 0.58 | % |
| pH | 6.5 | 7.4 | 6.3 | 6.4 | - |
| Cation exchange capacity | 27.0 | 27.0 | 19.0 | 20.0 | cmol/kg |
| Base saturation | 83.0 | 84.0 | 79.0 | 83.0 | % |

Table S2 Annual and seasonal climate characteristics of Weining and Shiqian sampling sites

| **Parameter** | **Weining** | **Tongren** | **Unit** |
| --- | --- | --- | --- |
| Elevation | 2236.0 | 418.0 | m |
| Mean temperature | 12.61 | 18.6 | ℃ |
| Mean maximum temperature | 19.09 | 24.14 | ℃ |
| Mean minimum temperature | 8.3 | 14.73 | ℃ |
| Maximum temperature extreme | 29.28 | 40.78 | ℃ |
| Minimum temperature extreme | -5.72 | -2.78 | ℃ |
| Days with mean temperature ≥18℃ | 93 | 182 | days |
| Days with mean temperature ≤0℃ | 21 | 4 | days |
| Annual precipitation | 557.78 | 1006.86 | mm |
| Maximum daily precipitation | 29.97 | 78.49 | mm |
| Number of precipitation days | 139 | 124 | days |
| Precipitation (Jun–Aug) | 236.73 | 420.62 | mm |
| Mean temperature (Mar–May) | 7.62 | 18.48 | ℃ |
| Mean temperature (Jun–Aug) | 13.76 | 26.94 | ℃ |
| Mean temperature (Sep–Nov) | 18.92 | 20.48 | ℃ |


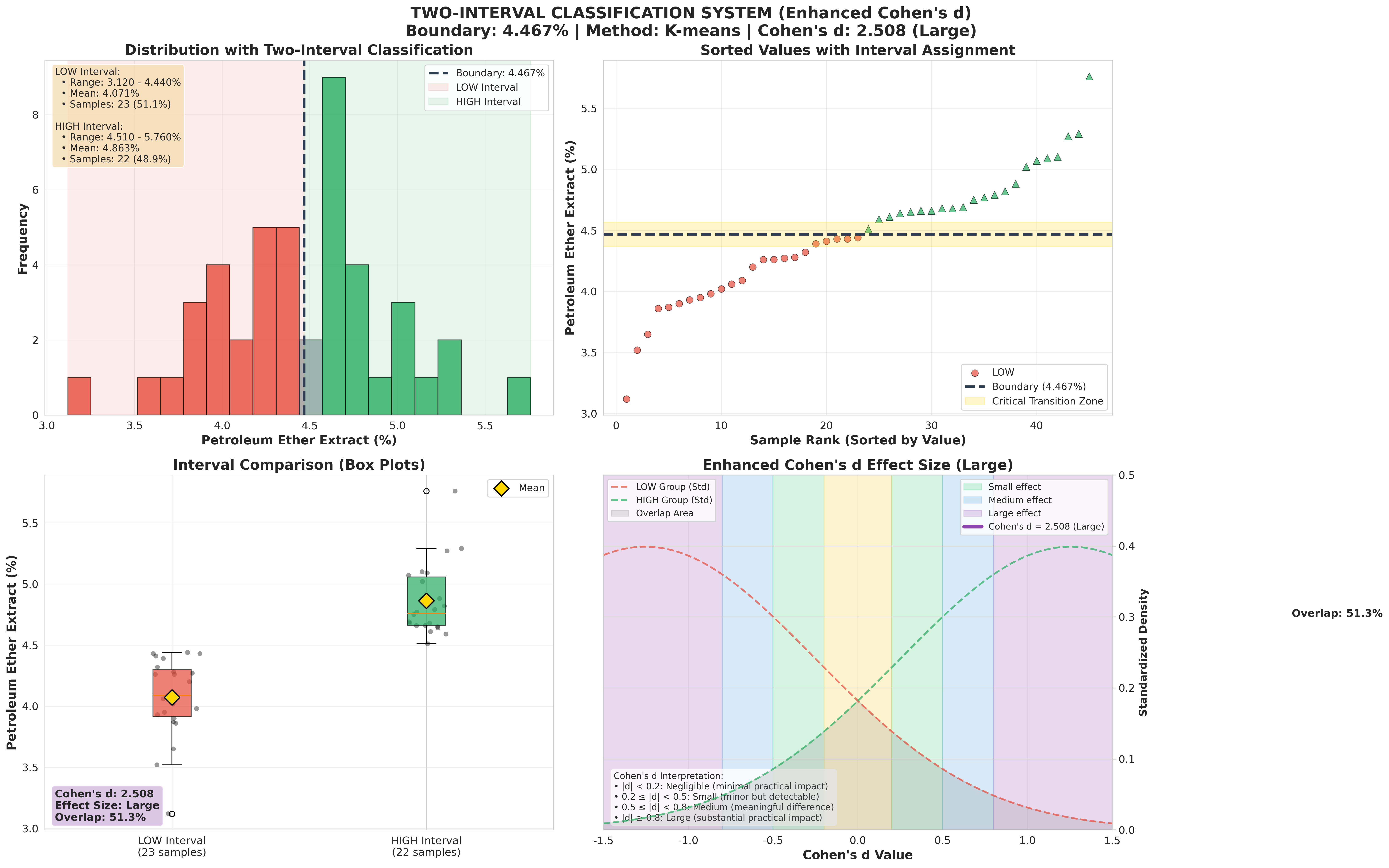


Figure S2 Two-interval classification of petroleum ether extract content in WN region based on data-driven boundary optimization


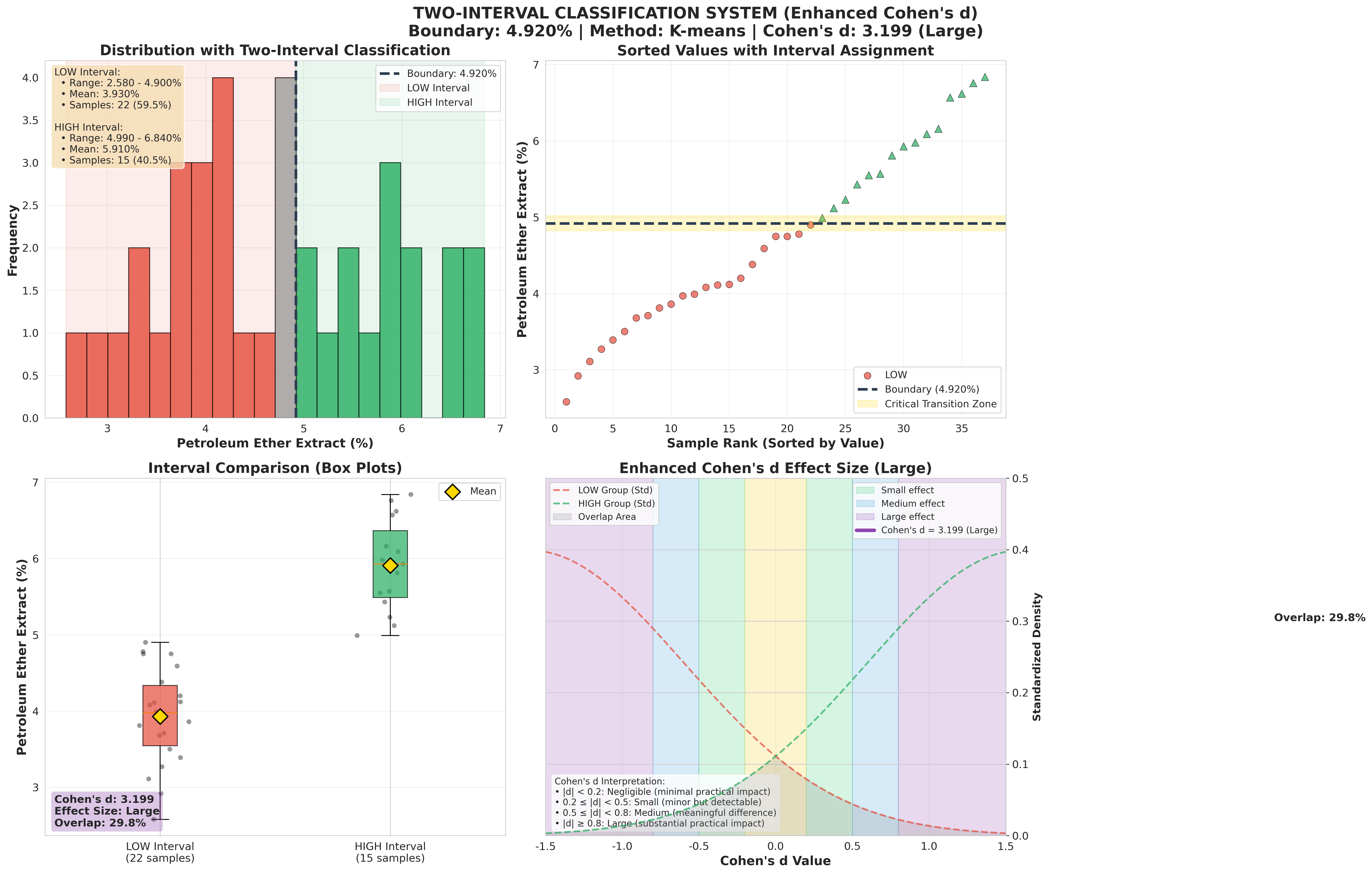


Figure S3 Two-interval classification of petroleum ether extract content in TR region based on data-driven boundary optimization

Table S3 Correlation between selected metabolites and tobacco leaf appearance traits

| **Metabolite** | **Trait** | **Softness** | **L*** | **a*** | **b*** | **Starch** |
| --- | --- | --- | --- | --- | --- | --- |
| Arteannuin | r | -0.13 | -0.91* | 0.86* | 0.15 | 0.84* |
|  | *p* | 0.69 | 0.69 | 0.69 | 0.69 | 0.69 |
| Isoferulic acid | r | 0.13 | 0.13 | 0.13 | 0.13 | 0.13 |
|  | *p* | 0.69 | 0.69 | 0.69 | 0.69 | 0.69 |
| Marmesin | r | -0.13 | -0.13 | -0.13 | -0.13 | -0.13 |
|  | *p* | 0.69 | 0.69 | 0.69 | 0.69 | 0.69 |
| p-Hydroxy-cinnamic acid | r | -0.13 | -0.13 | -0.13 | -0.13 | -0.13 |
|  | *p* | 0.69 | 0.69 | 0.69 | 0.69 | 0.69 |
